# Acetate trafficking in the heart: carnitine acyltransferases matter

**DOI:** 10.14814/phy2.12997

**Published:** 2016-10-05

**Authors:** Victor Zammit, Arduino Arduini

**Affiliations:** ^1^ Translational MedicineWarwick Medical SchoolUniversity of WarwickCoventryUK; ^2^ R&D DepartmentCoreQuest SaglMannoSwitzerland

Dear Editor,

The use of hyperpolarized ^13^C magnetic resonance to study intermediary metabolism is increasing rapidly, since it allows in vivo noninvasive investigation of dynamic metabolic and physiological processes that were previously experimentally inaccessible. A recent study published in the Journal by Koellish and colleagues used the technique to address the fate of hyperpolarized ^13^C‐labeled acetate in STZ‐induced diabetic rats (Koellisch et al. 2015a). While the use of this advanced technology is welcome, a basic misinterpretation of the cellular metabolic fate of acetate is apparent in the paper (Koellisch et al. 2015a). In particular, the authors have seriously misrepresented the mechanism of transfer of the acetate moiety across the mitochondrial inner membrane, confusing it with the one utilized by *long‐chain* fatty acids, in which the carnitine *palmitoyl*transferases (CPT1 and CPT2) and the inner membrane carrier carnitine acylcarnitine translocase (CACT) are crucial (Zammit 1999). In addition, because of this error, the authors state that carnitine *acetyl*transferase (termed CAT1 by the authors) is sensitive to malonyl‐CoA inhibition. This is not tenable, as malonyl‐CoA inhibition relates solely to carnitine *palmitoyl*transferases (CPT1A, CPT1B), and not to carnitine acetyltransferase. This basic misunderstanding of acetate metabolism is also apparent in previous work published by these authors (Koellisch et al. 2015b).

Carnitine acetyltransferase (generally referred to as CRAT or CAT) has mitochondrial and peroxisomal isoforms (which are *not* referred to as 1 or 2) and are *not* malonyl‐CoA sensitive. The formation of the latter is a diversion of acetyl‐CoA formed by *β*‐oxidation of fatty acids (and other cellular fuels) from oxidation in the tricarboxylic acid cycle (Zammit et al. 2009). The pathway: acetate → acetyl‐CoA → acetylcarnitine cannot be equated with the “rate of fatty acid oxidation”, especially when artificially high levels of acetate are imposed experimentally. The formation of acetylcarnitine has an important part to play in the regulation of intramitochondrial acetyl‐CoA levels, and, therefore, the balance between glucose and fatty acid utilization by tissues (Zammit et al. 2009). But, rationalization of this role requires knowledge of the identity, properties and roles of the different members of the carnitine acyltransferase family of enzymes, and specifically, an appropriate distinction between those that catalyze the utilization of carnitine esters with different acyl chain lengths.

In addition, it is noteworthy that parenteral administration of very high acetate dosages, as used by Koellish et al., can be calculated to lead to a plasma exposure of approximately 10 mmol/L, whereas physiological acetate levels are usually very much lower in the plasma of mammals (<0.1 mmol/L) (Veech 1988). Therefore, the protocol used may have significantly, and nonphysiologically, perturbed the cellular, and particularly, the intramitochondrial acetyl‐CoA pool (Taegtmeyer et al. 1980). Furthermore, the severity of the diabetic state induced by the experimental model (streptozotocin injection) appears to have been very variable, as the reported blood glucose levels ranged from 10 to 30 mmol/L (Koellisch et al. 2015a). Assuming that glycemic levels were obtained in fasted animals (not stated in the manuscript) such variability may further hamper the interpretation of the tracer data obtained.
